# Intracellular and extracellular rhomboid shaped crystalline inclusions in a case of IgG lambda restricted plasma cell myeloma: a case report and review of the literature

**DOI:** 10.1186/1746-1596-5-6

**Published:** 2010-01-21

**Authors:** Andres Matoso, Tina Rizack, Diana O Treaba

**Affiliations:** 1 Department of Pathology and Laboratory Medicine, Rhode Island Hospital, Providence, RI, USA; 2 Department of Medicine Division of Hematology and Oncology, Rhode Island Hospital, Providence, RI, USA; 3 Alpert Medical School of Brown University, Providence, RI, USA

## Abstract

The presence of crystalline inclusions in plasma cell myeloma is a rare phenomenon and cases have been reported with rod, needle, and rectangular shaped crystals. Here, we present a case of IgG lambda restricted plasma cell myeloma with rhomboid shaped intracellular crystalline inclusions and extracellular crystal depositions in the bone marrow. Since rhomboid crystal depositions can be seen in other clinical conditions such as pseudogout, this case invites consideration of plasma cell myeloma in the differential diagnosis of patients with rhomboid crystalline deposition in the bone marrow and in sites/organs other than the bone marrow.

## Background

Plasma cell myeloma accounts for approximately one percent of all cancers and slightly more than 10 percent of hematologic malignancies in the United States [1]. The disease stems from malignant transformation of plasma cells with frequent overproduction of immunoglobulins. Its clinical presentation with intracellular and extracellular crystalline inclusions is a rare but recognized phenomenon [2-4]. Crystalline inclusions with rod, needle, and rectangular shapes have been associated with free kappa or with IgA, IgD, IgG, and kappa light chain gammopathies [5-7]. Additionally, crystalline structures have been described in the cytoplasm of plasma cells in a patient with adult Fanconi syndrome and plasma cell myeloma [5]. It has been determined that crystalline structures are of immunoglobulin origin and are found not only in plasma cells but also in other hematopoietic cells [6]. Here, we present a case of IgG lambda restricted plasma cell myeloma with rhomboid intracytoplasmic crystalline inclusions and extracellular crystal deposition.

## Case Presentation

The patient is a 72 year-old male who was referred to a hematologist for work-up of anemia and leukopenia. He has had a gradual and persistent decrease in his hemoglobin levels during the past 3 years (13.6 G/DL in average; reference values 13.5-16.0 G/DL) associated with mild leukopenia (3.4 × 10^3^/μL; reference values 3.5-11.0 × 10^9^/L). Additionally, a year prior to the current presentation, the patient developed intermittent episodes of confusion and memory loss. The latest complete blood count was remarkable for hemoglobin of 12.1 g/dL and white blood cell count of 3.3 × 10^3^/μL. The protein level in a 24-hour urine sample was elevated to 810 mg/24 hs (reference values 42-225 mg/24 hs). BUN and creatinine were within the normal range. Serum protein electrophoresis revealed a hypogammaglobulinemia pattern with monoclonal gamma paraprotein (0.83 g/dL). Serial radiographs of the calvarium, cervical, thoracic, lumbar spine, as well as bilateral humeri and femora did not show lytic lesions.

**Figure 1:**
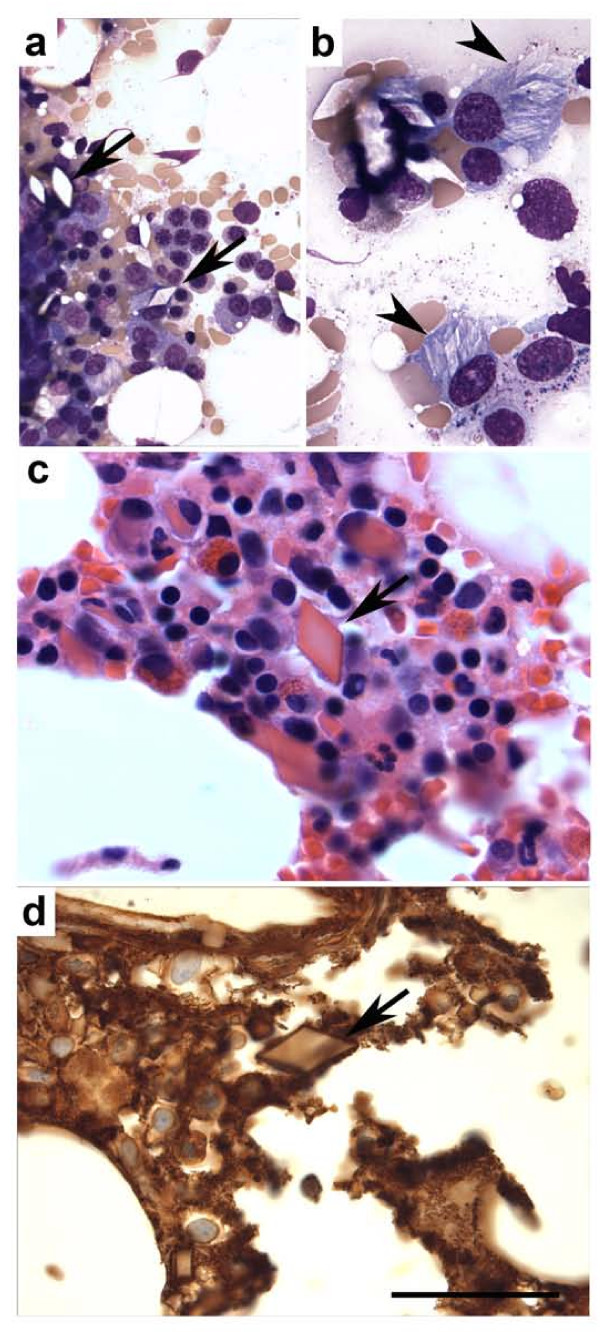
**Plasma cell myeloma with intracytoplasmic and extracellular rhomboid crystalline inclusions**. (a and b) Bone marrow aspirate stained with Wright-Giemsa. Note abundant rhomboid crystals present in the extracellular space (a; arrows) and intracytoplasmic in plasma cells (b; arrow-heads). (c) H&E stain of bone marrow biopsy section showing prominent rhomboid crystals (arrow). (d) Immunohistochemistry for lambda-chain highlights monotypic plasma cells with positive stain of the extracellular crystals (arrow). Bar 200 μm (a), 20 μm (b), and 50 μm (c and d).

Both, the bone marrow biopsy and aspirate had normocellular bone marrow with trilineage hematopoiesis, slightly decreased myeloid and erythroid series and an increased population of plasma cells, 27% plasma cells in the 500 cell count aspirate differential (normal range up to 3%). Megakaryocytes were present in adequate number and had an unremarkable morphology. The plasma cell population included a subset with round conspicuous nucleoli and intracytoplasmic, single or multiple, nonbirefringent translucent crystalline structures with rhomboid shapes. Scattered free extracellular rhomboid shaped crystals were also noted (Figure 1a, 1b and 1c). Flow cytometry immunophenotypic analysis performed on bone marrow aspirate sample identified a monotypic, cytoplasmic lambda light chain restricted CD38+, CD138+ plasma cell population with dim CD45+, CD56+ and CD117+ expression. By immunohistochemistry, there were approximately 25-27% CD138+ plasma cells, which in a large subset expressed immunoglobulin lambda light chain restriction, and a subset was also IgG restricted. The crystals were weakly Ig lambda positive (Figure 1d). A Congo red stain to evaluate for possible amyloid deposition was negative.

Due to underlying cognitive deficits a decision was made to initiate low dose lenalidomide therapy at 15 mg daily for 21 out of every 28 days. Within the first week of medical treatment the patient developed a pruritic rash on his bilateral arms which disappeared after seven days but reappeared with increasing severity and more forgetfulness with the second cycle of lenalidomide. The patient was then switched to thalidomide at 100 mg/day but due to fatigue the dose was decreased to 50 mg daily which the patient continues to tolerate well. Dexamethasone was held due to clinical concern for increasing of patient's cognitive deficits with plans of a trial if he tolerates thalidomide. His paraprotein levels stayed relatively stable with the lenalidomide, 0.83 g/dl at diagnosis to 0.81 g/dl 6 weeks later, and a mild decrease to 0.75 g/dl 4 weeks after beginning thalidomide.

## Discussion

Plasma cell crystalline inclusions with rod, rectangular, and needle-like shapes have been described in cases of multiple myeloma and they are believed to be due to accumulation of cytoplasmic immunoglobulins secondary to a block in the protein synthetic pathway [2,5,8]. Herein we present a case of plasma cell myeloma with rhomboid intracytoplasmic crystalline inclusions and extracellular crystals deposition. It is of note that it has been postulated that the presence of crystalline inclusions may indicate a nonprogressive clinical course of the disease [8]. However, since this morphological pattern of presentation of multiple myeloma is very rare, its prognostic significance is currently largely unknown. Yet, it is important to consider this association as part of the differential diagnosis in a patient with plasma cell myeloma and, for example, arthritis with crystal deposition. Rectangular, rod-like and rhomboid-like crystals in the joints could be seen in cases of calcium pyrophosphate dihydrate (CPPD) crystal deposition disease or pseudogout. In plasma cell myeloma, a rheumatoid-like seronegative polyarticular erosive arthropathy is an unusual but reported presentation characterized by crystal deposition of cryoglobulins in the synovium and several other tissues [9]. The patient presented here did not report articular symptoms for which we could not yet establish a connection between crystalline deposits in the bone marrow and the synovium.

Renal parenchyma damage in patients with plasma cell myeloma is frequent and well characterized and can involve crystal depositions. Renal biopsy can show intracytoplasmic crystalline inclusions most frequently located in the distal tubule, but that can also be seen in the proximal tubule [10]. So far, the patient presented here has not suffered renal insufficiency and a renal biopsy is not available. Therefore, an association between bone marrow and renal parenchyma crystalline deposition could not be made.

The skin and subcutaneous tissue could also be a site of crystalline depositions in patients with plasma cell myeloma [11,12]. In such cases, crystalloid structures have been identified within histiocytes and the entity interpreted as a cutaneous crystal storing histiocytosis [11]. Interestingly, a case of bilateral ecchymoses and corneal crystal deposition have been reported as initial presentation of plasma cell myeloma in a 55 year-old man [13]. This patient presented with amyloidosis and Bence-Jones proteins but crystalline deposits in the bone marrow were not reported.

The differential diagnosis in cases of plasma cell intracytoplasmic crystalline inclusions should also include reactive processes, since it has been shown to occur in cases of helicobacter associated gastritis [14]. In the case presented here, a reactive process is ruled out due to the monotypic nature of the plasma cell population.

Finally, crystalline inclusions similar to those seen in plasma cell myeloma have been described in cases of granulocytic sarcoma [15]. Electron microscopy demonstrated homogeneously dense, bipyramidal structures, similar to Charcot-Leyden crystals. Should the morphology be equivocal, immunophenotypical and molecular studies might be necessary to yield the correct diagnosis.

Of note, somewhat unusual in our patient is the clinical presentation with cognitive deficits. While this could be merely a coincidental event, a possible association with patient's myeloma cannot be completely excluded based on the current data available. Histopathological evaluation of the central nervous system and/or future case reports of similar clinical and histopathological presentations should help to understand whether such a connection exists.

In summary, this case of plasma cell myeloma with rhomboid crystalline inclusions invites review and consideration of plasma cell myeloma in cases presenting with intracytoplasmic crystalline inclusions and extracellular crystals depositions. The literature suggests that plasma cell myeloma and crystal deposition can occur in different sites such as joints, kidneys, skin and cornea in addition to bone marrow. None of the cases reported so far presented with crystal deposition in the bone marrow and in other sites.

## Conclusions

The case presented here illustrates an unusual finding in a patient with plasma cell myeloma. This case and the review of similar cases presented in the literature suggest that extracellular rhomboid crystals in the bone marrow and extramedullary sites such as articular spaces or renal parenchyma should raise suspicion of a plasma cell neoplasm, yet other differential diagnoses such as granulocytic sarcoma or reactive processes should also be considered.

## Consent

Written informed consent was obtained from the patient for publication of this case report and any accompanying images. A copy of the written consent is available for review by the Editor-in-Chief of this journal.

## Competing interests

The authors declare that they have no competing interests.

## Authors' contributions

AM was involved in the histopathology evaluation, preparing the materials, literature search and drafting the manuscript. TR supplied the relevant clinical details. DOT outlined the general concept, interpreted the histopathology, and revised the manuscript. All authors have read and approved the present manuscript.
